# Identification of a Receptor for Extracellular Renalase

**DOI:** 10.1371/journal.pone.0122932

**Published:** 2015-04-23

**Authors:** Ling Wang, Heino Velazquez, John Chang, Robert Safirstein, Gary V. Desir

**Affiliations:** 1 Department of Medicine, VACHS, Yale University, New Haven, Connecticut, United States of America; 2 Renal Division, Renji Hospital, Shanghai Jiaotong University School of Medicine, Shanghai, China

## Abstract

**Background:**

An increased risk for developing essential hypertension, stroke and diabetes is associated with single nucleotide gene polymorphisms in renalase, a newly described secreted flavoprotein with oxidoreductase activity. Gene deletion causes hypertension, and aggravates acute ischemic kidney (AKI) and cardiac injury. Independent of its intrinsic enzymatic activities, extracellular renalase activates MAPK signaling and prevents acute kidney injury (AKI) in wild type (WT) mice. Therefore, we sought to identity the receptor for extracellular renalase.

**Methods and Results:**

RP-220 is a previously identified, 20 amino acids long renalase peptide that is devoid of any intrinsic enzymatic activity, but it is equally effective as full-length recombinant renalase at protecting against toxic and ischemic injury. Using biotin transfer studies with RP-220 in the human proximal tubular cell line HK-2 and protein identification by mass spectrometry, we identified PMCA4b as a renalase binding protein. This previously characterized plasma membrane ATPase is involved in cell signaling and cardiac hypertrophy. Co-immunoprecipitation and co-immunolocalization confirmed protein-protein interaction between endogenous renalase and PMCA4b. Down-regulation of endogenous PMCA4b expression by siRNA transfection, or inhibition of its enzymatic activity by the specific peptide inhibitor caloxin1b each abrogated RP-220 dependent MAPK signaling and cytoprotection. In control studies, these maneuvers had no effect on epidermal growth factor mediated signaling, confirming specificity of the interaction between PMCA4b and renalase.

**Conclusions:**

PMCA4b functions as a renalase receptor, and a key mediator of renalase dependent MAPK signaling.

## Introduction

Renalase is a novel secretory flavoprotein oxidase [1–4]. Single nucleotide polymorphisms present in the gene are associated with hypertension, cardiac disease and diabetes [3, 5–8]. Renalase’s crystal structure has been solved [9], and while it was initially described as having NADH oxidase activity, it was recently determined that it metabolizes 2- and 6-dihydroNAD(P), which are isomeric forms of β-NAD(P)H [3, 10]. The administration of renalase in wild type (WT) mice lowers plasma catecholamines and systemic blood pressure. Renalase deletion in mice (renalase KO) raises catecholamine levels and blood pressure [11]. Gene deletion also aggravates acute ischemic kidney (AKI) [12], and cardiac injury [11]. Recombinant renalase prevents ischemic injury in wild type mice [12].

Although renalase’s crystal structure has been solved, the molecular details of its actions remain uncertain. We recently reported that renalase promotes cell and organ survival through a receptor-mediated process that is independent of its intrinsic enzymatic activities [13]. We also identified the critical region of renalase molecule that mediates its cytoprotective effects [13], and showed that a 20 amino acid renalase peptide (RP-220, aa 220–239: CIRFVSIDNKKRNIESSEIG), which is conserved in all known isoforms but is devoid of any detectable oxidase activity, was equally effective as intact renalase protein at protecting HK-2 cells and WT mice against toxic and ischemic injury.

RP-220 and recombinant renalase rapidly activated protein kinase B (AKT), extracellular signal-regulated kinase (ERK), and mitogen activated protein kinase (p38) mitogen activated protein kinases, suggesting that RP-220 is the region of the renalase protein critical for interaction with its cognate receptor. In this work, using RP-220 as a probe, we have identified the plasma membrane calcium ATPase PMCA4b as a renalase receptor, which mediates renalase-dependent cell signaling and cytoprotection.

## Methods

All studies involving animals were approved by the IACUC of Veterans Administration Connecticut Health Care System (VACHS). We have used the commercially available human kidney embryonic cell line, HK-2 in prior studies [12, 13], and the studies described here were approved by the Research Safety Committee of VACHS.

### Synthesis and analysis of renalase and renalase peptides

**Fig 1:**
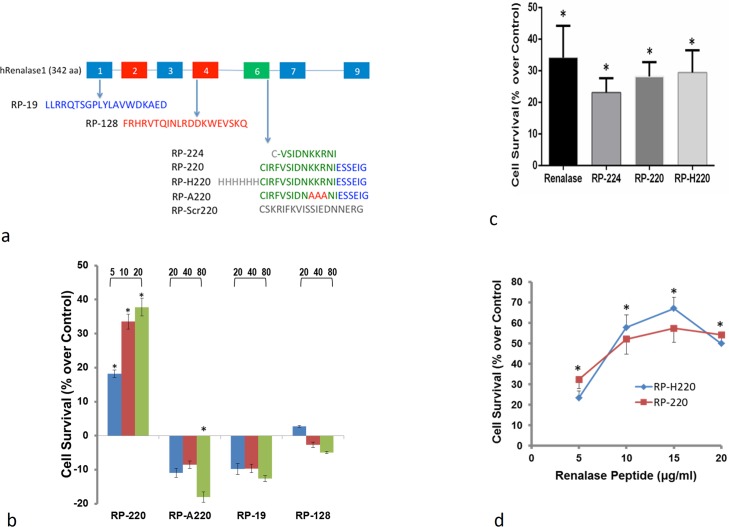
Renalase peptides demonstrate protective effects. A, amino acid sequence of renalase peptides: RP-Scr220: scrambled RP-220. B, Effect of renalase peptides on survival of HK-2 cells exposed to 20μM cisplatin for 24 hrs;. cell survival is depicted as % change in survival compared to that in cisplatin-treated HK-2 cells without renalase peptides; cell survival measured by the WST-1 method, RP-A220: mutated RP-220, and RP-19 and RP-128: control peptides; peptide concentration (μg/ml) indicated in top line; n = 4, * = p<0.05. C, Comparison of protective effect of recombinant renalase, RP-224, RP-220, and RP-H220 on survival of HK-2 cells exposed to 20μM cisplatin for 24 hrs; cell survival is depicted as % change in survival compared to that in cisplatin-treated HK-2 cells without renalase peptides; n = 4, * = p<0.05. D, Dose response curve for RP-220 and RP-H220; HK-2 cells exposed to 20μM cisplatin for 24 hrs; cell survival is depicted as % change in survival compared to that in cisplatin-treated HK-2 cells without renalase peptides; n = 4, * = p<0.05.

Renalase peptides (sequences shown in Fig 1A) were acetylated at the amino terminus and purified to 98% homogeneity (United Peptides, Herndon, VA). Recombinant renalase was synthesized as previously described [4]. Renalase expression was detected using an anti-renalase monoclonal antibody generated against the renalase peptide RP-220 (amino acid 220–239 of hRenalase1) [12].

### Renalase receptor cross linking and identification

Potential disulfide bonds formed by the single, amino terminal cysteine present in renalase peptide 220 (RP-220), and in the scrambled renalase peptide (RP-Scr220, control peptide) (Fig 1A) were disrupted using an immobilized reducing column (#77701, Pierce Biotechnology, Rockford, IL). The reduced peptides were recovered by elution, and the concentration of free sulfhydryl groups was estimated using the Ellman’s Reagent (#22582, Pierce Biotechnology, Rockford, IL).

Reduced RP-220 and RP-Scr220 were conjugated to a tri-functional cross-linker, Mts-Atf-Biotin (#33093, Pierce Biotechnology, Rockford, IL) according to the manufacturer’s instructions. Conjugation was achieved through the sulfhydryl-reactive methanethiosulfonate (Mts) moiety, and the spacer arm between Mts, and the photoactivatable tetrafluorophenyl azide (Atf) moiety was 11 Å. Coupling efficiency was estimated by dot blot using streptavidin-HRP to measure biotin incorporation (#21130, Pierce Biotechnology, Rockford, IL). The labeled probes were protected from light and stored in 50 μl aliquots, at -80°C until use.

HK-2 cells, obtained from American Type Culture Collection (ATCC, Manassas, VA) were grown at 37°C to 80% confluence in 150 mm dishes in DMEM/F12 media supplemented with glutamine, 10%FBS, antibiotics, and 5% CO2. Cells were cooled to 4°C to prevent probe internalization, and then incubated for 16 hrs with 50 μg of either Mts-Atf-Biotin labeled RP220 or RP-Scr220. Cross-linking of the probes was initiated by exposing the cells to ultraviolet light for five minutes using a Stratalinker 2500 (Stratagene, Agilent Technologies, Santa Clara, CA).

The cells were suspended in phosphate buffered saline (PBS) with protease inhibitors (Complete Ultra # 05892953001, Roche Diagnostics, Indianapolis, IN) and subjected to 3 cycles of freeze-thawing. The membrane fraction was collected by centrifugation (180,000g for 1 hour) and solubilized in RIPA buffer (20 mM Tris-HCl pH 7.5, 150 mM NaCl, 1 mM Na_2_EDTA, 1 mM EGTA, 1% NP-40, 1% sodium deoxycholate, 2.5 mM sodium pyrophosphate, 1 mM glycerophosphate, 1 mM Na_3_VO_4_, 1μg/ml leupeptin) (Cell Signaling Technologies, Danvers, MA) for 4 hours at 4°C

The biotinylated proteins were purified using streptavidin agarose resin (Pierce Biotechnology, Rockford, IL) according to the manufacturer’s instructions. The proteins were separated by SDS PAGE using a 4–20% gradient gel (Bio-Rad, Hercules, CA) and stained by Western-blotting using streptavidin-HRP to identify proteins bands found predominantly cross-linked to the renalase bait RP-220. These bands and the corresponding bands from the samples cross-linked to the control bait RP-Scr220 were cut out of coomassie blue stained gels, and individual proteins were identified by mass spectrometry (Yale Keck Biotechnology Resource Laboratory, S1 Methods). Plasma membrane protein(s) consistently present in the samples cross-linked to RP-220, and absent from those cross-linked to the control bait RP-Scr220 were evaluated further.

### Downregulation of PMCA4b expression using siRNA

ATP2B4 (PMCA4b) specific siRNAs and non-targeting controls (SMARTpool ON-TARGETplus ATP2B4 siRNA, L-006118-00-0005) were purchased from Thermo Fischer Scientific (Waltham, MA), and transfected into HK-2 cells using Lipofectamine 2000 (Invitrogen, Life Technologies Grand Island NY). HK-2 cells were grown to 70% confluence in DMEM/F12 medium supplemented with 10%FBS. PMCA4b protein expression was assessed by western immunoblot using an anti-PMCA4 mouse monoclonal antibody (#H00000493-M07, Novus Biologicals, Littleton, CO).

### Detection of endogenous co-expression of PMCA4b and renalase

HK-2 cells, which highly express renalase and PMCA4b endogenously, were fixed, permeabilized, and incubated with a goat polyclonal anti-renalase antibody (# AF5350, R&D Systems, Minneapolis, MN), and an anti-PMCA4 mouse monoclonal antibody (#H00000493-M07, Novus Biologicals, Littleton, CO) for 2 hours at room temperature. Following the application of two labeled secondary antibodies (Alexa488-rabbit anti-goat to detect renalase and Alexa555-goat anti-mouse to detect PMCA4, Molecular Probes, Life Technologies Grand Island NY), cells were then examined using a Zeiss LSM 510 confocal imaging system.

### Co-Immunoprecipitation of endogenous PMCA4b and renalase

HK-2 cells were lysed using ice-cold RIPA buffer (Cell Signaling Technologies, Danvers, MA), and the lysate was centrifuged 14,000 g for 15 min. The supernatant was collected and incubated with protein A/G agarose beads (Santa Cruz Biotechnology, Santa Cruz, CA) to reduce non-specific binding. Immunoprecipitation was carried out using A/G agarose beads and either goat polyclonal anti-renalase antibody or anti-PMCA4 mouse monoclonal antibody, and proteins were visualized by western blotting.

### Gene expression analysis

Total RNA from tissue samples was extracted using RNeasy Plus kit (Qiagen, Valencia, CA) and converted into cDNA using an Omniscript RT kit (Qiagen, Valencia, CA). PMCA4b mRNA in kidney was assessed in renalase KO mice by real-time PCR and normalized to the corresponding levels in WT samples (defined as 1.0). Target cDNA was amplified using Qiagen Dr_atp2b4_1_SG QuantiTect primer assay and Platinum SYBR Green qPCR superMix-UDG. Standard cycling conditions were run with a Step-One-Plus real time PCR system (Applied Biosystems), and the resulting Ct values analyzed using the 2-ΔΔCT method.

### In vitro model of cisplatin toxicity

HK-2 cells obtained from ATTC (Manassas, VA USA) were cultured in DMEM/F12 supplemented with glutamine, 10%FBS and antibiotics, and were maintained at 37°C in 5% CO_2_. Cells were exposed to cisplatin (20 μM) in the presence or absence of recombinant renalase or renalase peptides for 24 hrs, and cell viability was assessed by the WST1 method (Roche Applied Science, Germany).

To examine renalase dependent MAPKs signaling, cells treated with RP-220 (15ug/ml) or EGF (100ng/ml) as a positive control were harvested in RIPA buffer (20 mM Tris-HCl, pH 7.5, 150 mM NaCl, 1 mM Na_2_EDTA, 1 mM EGTA, 1% NP-40, 1% sodium deoxycholate, 2.5 mM sodium pyrophosphate, 1 mM α-glycerophosphate, 1 mM Na_3_VO_4_, 1 μg/ml leupeptin) supplemented with a protease and phosphatase inhibitor cocktail (Roche Applied Science, Germany). Proteins were separated by SDS-PAGE and immunoblotting was carried out using the following antibodies: anti-renalase monoclonal antibodies [4, 12], and antibodies specific for total and phosphorylated ERK, p38, and JNK (Cell Signaling Technology, MA, USA). The following inhibitors were obtained from Sigma-Aldrich Corp (St. Louis, MO): U0126 (1,4-diamino-2,3-dicyano-1,4-bis[2-aminophenylthio]) for ERK1/2, and SB203580 (4-[4’-Fluorophenyl]-2-[4’-methylsulfinylphenyl]-5- [4’-pyridyl]-imidazole) for p38 α-β.

## Statistical analysis

When appropriate, the Kruskal–Wallis one-way analysis of variance by ranks was used to evaluate statistical significance. When the Kruskal–Wallis test revealed statistical significance, the Mann-Whitney test was used for pairwise comparisons. All data are mean ± SEM, and values of *P*<0.05 were accepted as a statistically significant difference. Statistical analysis was carried out using GraphPad Prism (GraphPad Software, Inc.).

## Results

### Protection by RP-220 against cisplatin toxicity linked to p38 MAPK activation

We have previously shown that in renalase KO mice renalase deficiency worsens ischemic AKI, while the administration of recombinant renalase significantly attenuates AKI [12]. Additional studies indicate that the protective effect of renalase against ischemic and cisplatin AKI does not depend on the enzymatic activity of renalase, but rather is mediated by the interaction of renalase or short renalase peptides (RP-224, RP-220 and RP-H220, Fig 1A) with a receptor(s). A scrambled renalase peptide, RP-Scr220 did not activate MAPK signaling, and failed to protect cells [13]. The protective effects of renalase peptides corresponded to the activation of intracellular signaling that promotes cell survival [13].

To determine if RP-220 could be used as probe to identify the receptor(s) for extracellular renalase, we examined its specificity by comparing its protective effect against cisplatin toxicity to that of mutated RP-220 (RP-A220), and of renalase peptides RP-19 and RP-128 (Fig 1A). Renalase peptides RP-19 and RP-128 were chosen because they contain putative ERK docking (D) domains (Motif Scan) [14]. RP-220 significantly protected HK-2 cells exposed to cisplatin for 24 hrs in a dose dependent manner (Fig 1B). Control peptides RP-19, RP-128, and RP-A220 did not improve cell survival, and at the highest concentration (80μg/ml) RP-A220 actually exacerbated cisplatin toxicity. Recombinant renalase (80 μg/ml), RP-224 (100 μg/ml), RP-220 (15 μg/ml), and RP-H220 (15 μg/ml) protected HK-2 cells to a similar degree (Fig 1C). The concentration dependent effects for RP-220 and RP-H220 indicate both peptides are equipotent at protecting HK-2 cells against cisplatin cytotoxicity, and RP-220 (15 μg/ml) was used for all subsequent studies (Fig 1D **)**.

**Fig 2:**
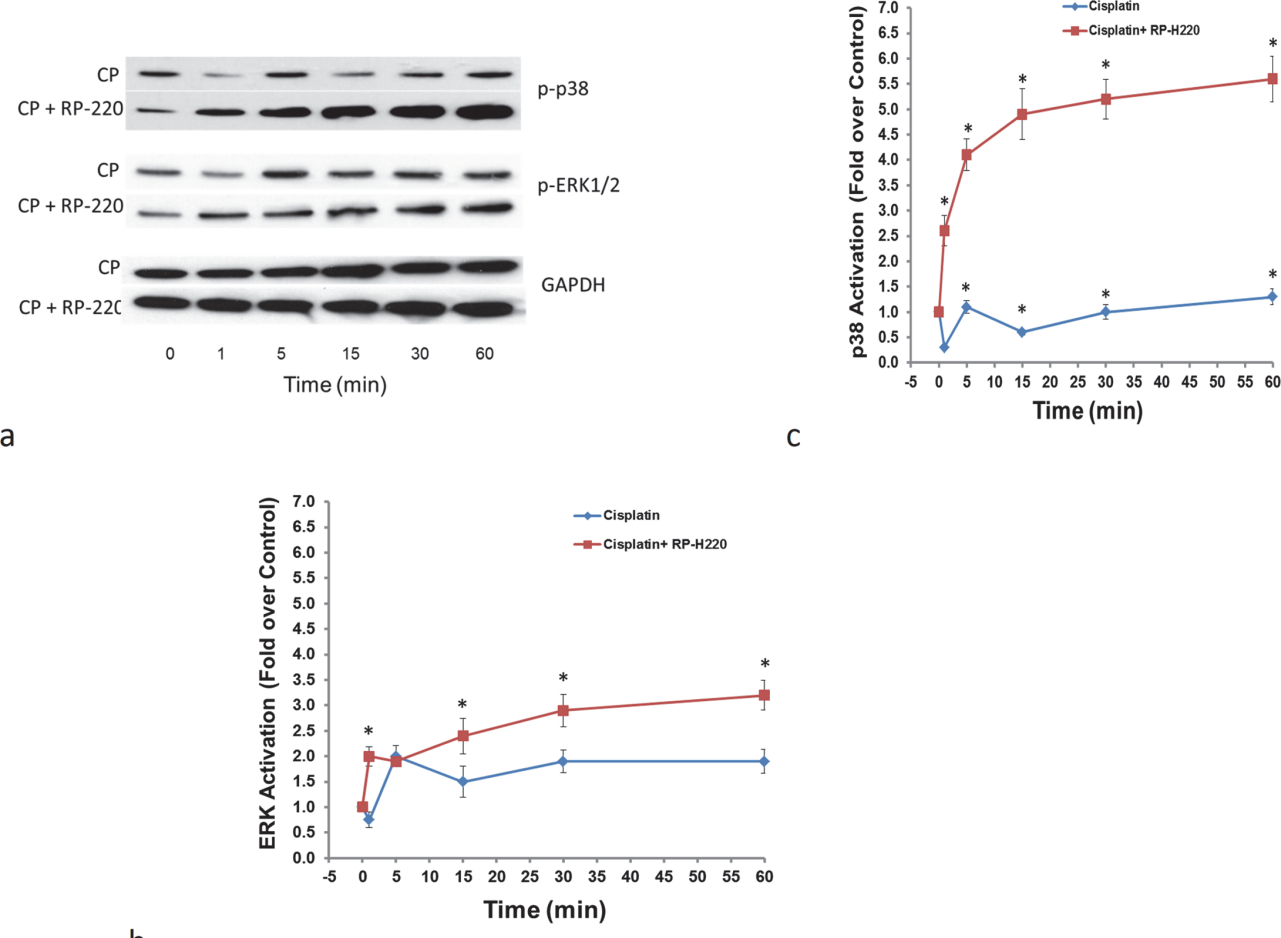
RP-220 alters the pattern of MAPK activation in cisplatin treated cells. A, MAPK phosphorylation with cisplatin alone (CP) and with CP and RP-220, representative study, p-p38: phosphorylated p38 MAPK; p-ERK: phosphorylated ERK. B, Quantification of ERK activation, signals normalized to glyceraldehyde 3-phosphate dehydrogenase (GAPDH) loading control; n = 3, * = P<0.05. C, Quantification of p38 activation, signals normalized to GAPDH loading control; n = 3, * = P<0.05.

We previously showed that both hRenalase1 and RP-220 treatments rapidly increased ERK and p38 phosphorylation in HK-2 cells [13]. To test if protection of HK-2 cells against cisplatin toxicity by RP-220 was linked to MAPK activation, we examined the patterns of MAPK signaling at early time points (1–60 min) in HK-2 cells exposed to cisplatin with and without RP-220. Cisplatin alone modestly increased ERK and p38 phosphorylation (Fig 2A–2C). This effect on p38 phosphorylation was markedly enhanced (5 fold) by the addition of RP-220 (Fig 2C). However, only a slight increase in ERK activation (0.5 fold) was noted with RP-220 over that elicited by cisplatin alone **(** Fig 2B). These results suggest that RP-220’s cytoprotective action may be due to its activation of p38 and not ERK.

**Fig 3:**
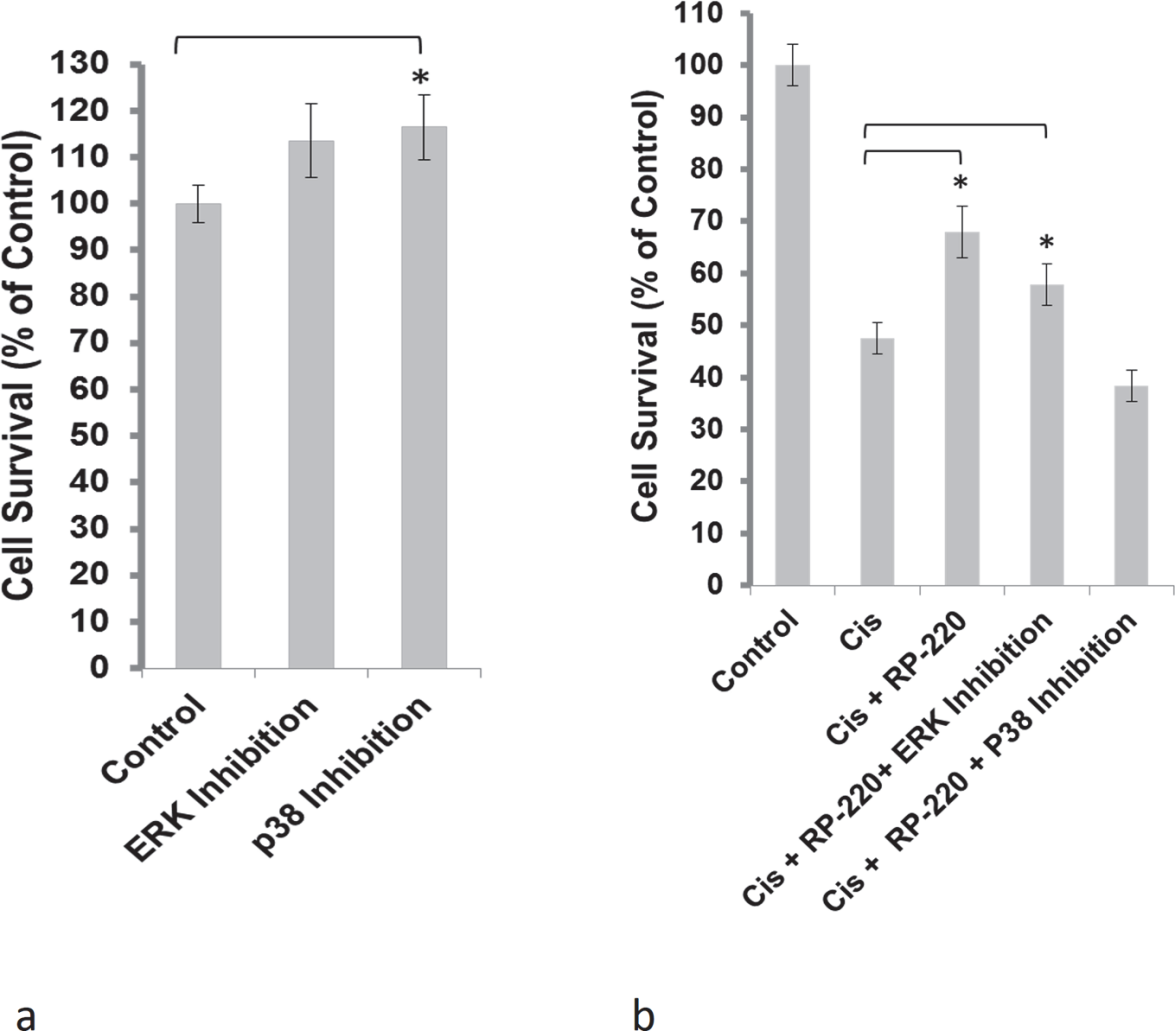
Inhibition of p38 abrogates the protective effect of RP-220. A, Inhibition of either p38 (SB203580) or ERK (U0126) did not adversely affect the survival of HK-2 cells measured using the WST-1 method; cell survival is depicted as % change in survival compared to that of untreated HK-2 cells; n = 4, * = P<0.05. B, Inhibition of p38 (10 μM SB203580)abrogated the protective action of RP-220 for HK-2 cells exposed to 20 μM cisplatin (Cis) for 24 hrs; n = 4, * = P<0.05.

To determine the relative importance of ERK and p38 as mediators of RP-220’s protective effect, their activities were chemically inhibited. Reducing the activity of ERK1/2 (U0126), or p38α-β (SB203580) was examined in HK-2 cells under control conditions. In control studies, ERK and p38 inhibition had no deleterious effect and not found to reduce HK-2 cell survival (Fig 3A). In HK-2 cells treated with both cisplatin and RP-220, ERK inhibition did not diminish the protection conferred by RP-H220 (Fig 3B). In marked contrast, p38 inhibition completely abrogated the RP-220’s protection (Fig 3B), thus providing strong support for the hypothesis that p38 is a key mediator of RP-220’s defense against cisplatin toxicity.

### Extracellular renalase binds to the plasma membrane Ca^++^-ATPase isoform PMCA4b

**Fig 4:**
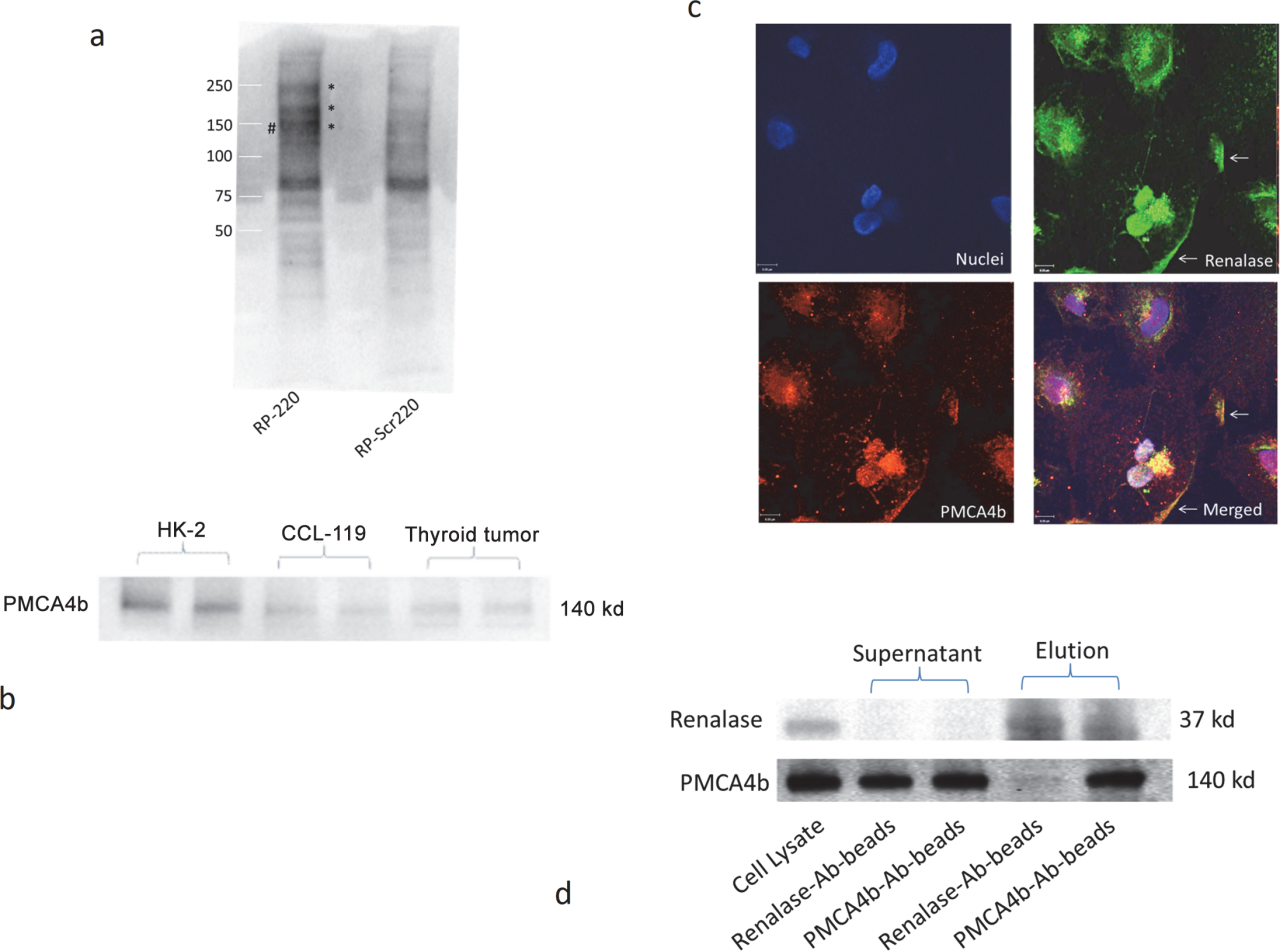
Identification of plasma membrane calcium ATPase isoform PMCA4b as a renalase binding protein. A, HK-2 cells incubated with either labeled RP-Scr220 or RP-220, biotin-labeled proteins purified using streptavidin column, separated by SDS-PAGE and visualized by western blot using streptavidin-HRP; * = regions evaluated by mass spectrometry in samples labeled with either RP-Scr220 or RP-220; # = RP-220 band containing the plasma membrane calcium ATPase isoform PMCA4b. B, Endogenous expression of PMCA4b in HK-2 cells, western immunoblot using isoform specific monoclonal; CCL-119: human leukemic cell line; thyroid tumor = human thyroid tumor cell line (ATCC, CRL-1803) 10 μg protein loaded in each lane. C, co-immunolocalization of PMCA4b and renalase in HK-2 cells, images acquired using a Zeiss laser scanning confocal microscope, scale bar = 9 μm; arrow = plasma membrane. D, Co-Immunoprecipitation of PMCA4b and renalase from HK-2 cell lysates; renalase-Ab-beads = renalase antibody coated beads; PMCA4b-Ab-beads = PMCA4b antibody coated beads.

RP-220 was used as a probe to identify a plasma membrane protein(s) that interact with extracellular renalase. The biotin label transfer method was used with the label transfer reagent Mts-Atf-Biotin, which was linked to the single cysteine located at the N terminus of RP-220. Labeled RP-220 was incubated with HK-2 cells for 24 hrs at 4°C to minimize internalization, and was cross-linked to the interacting protein(s) by exposure to UV light. The biotin-labeled proteins were purified using a streptavidin column and identified by mass spectrometry. The plasma membrane calcium-ATPase isoform, PMCA4b, was reproducibly cross-linked to RP-220 (Fig 4A). PMCA4b is abundantly expressed in HK-2 cells (Fig 4B) and co-localizes with renalase at the plasma membrane (arrows), and within the cytoplasm of HK-2 cells (Fig 4C).

In vitro interaction between endogenously expressed PMCA4b and RP-220 was evaluated by co-immunoprecipitation. Agarose beads coated with either PMCA4b or renalase antibody depleted whole cell lysates of renalase (Fig 4D, upper blot, lanes 1 vs 2 and 3), and renalase could be eluted from both sets of beads (Fig 4D, upper blot, lanes 4–5).). The blot was re-probed with an anti-PMCA4b antibody, and likewise, PMCA4b could be eluted from both sets of beads (Fig 4D, bottom blot, lanes 4–5). Of note, endogenous expression of PMCA4b is significantly higher than that of renalase (Fig 4D, upper and lower blots, lanes 1), and as a consequence less PMCA4b protein is eluted from beads coated with the renalase antibody than from those coated with the PMCA4b antibody. (Fig 4D, upper and lower blots, lanes 4 vs 5). In addition, PMCA4b coated beads completely depleted renalase from the supernatant suggesting that extracellular renalase is largely bound to PMCA4b.

### PMCA4b mediates renalase’s action on cell signaling and cytoprotection

**Fig 5:**
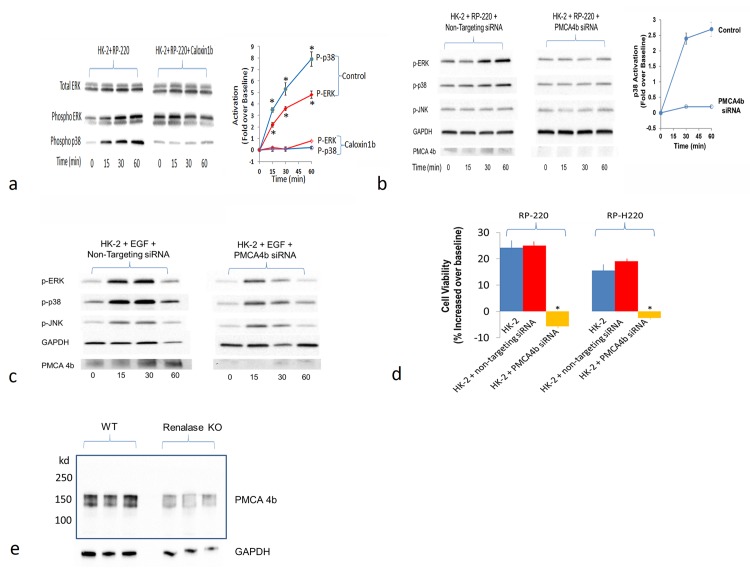
PMCA4b inhibition abrogates renalase peptide mediated MAPK signaling and cytoprotection. A, PMCA4b inhibition abrogates RP-220 mediated ERK and p38 signaling in HK-2 cells; caloxin1b = peptide inhibitor of PMCA4; *left panel*: RP-220 mediated ERK and p38 activation, phospho = phosphorylated, representative blot; *middle panel*: Inhibition of RP-220 mediated ERK and p38 activation by caloxin1b (100 μM); *right panel*: quantification of phosphorylated p38 (P-p38) and phosphorylated ERK (p-ERK), signals normalized to glyceraldehyde 3-phosphate dehydrogenase (GAPDH) loading control; n = 3, * = P<0.05. B, siRNA mediated inhibition of PMCA4b expression downregulates RP-220 mediated MAPK signaling; *left panel*: RP-220 mediated ERK and p38 activation in HK-2 cells transfected with non-targeting siRNA, p = phosphorylated, representative immunoblot; *middle panel*: Inhibition of RP-220 mediated ERK and p38 activation in HK-2 cells transfected with PMCA4b siRNA, representative blot; *right panel*: quantification of phosphorylated ERK (p-ERK), signals normalized to glyceraldehyde 3-phosphate dehydrogenase (GAPDH) loading control; n = 3, * = P<0.05. C, Lack of effect of siRNA mediated inhibition of PMCA4b expression on epidermal growth factor (EGF)- mediated MAPK signaling; *left panel*: EGF (100 ng/ml) mediated ERK, p38 activation and c-Jun N-Terminal Kinase (JNK) in HK-2 cells transfected with non-targeting siRNA, p = phosphorylated, representative blot; *right panel*: EGF-mediated ERK, p38 and JNK activation in HK-2 cells unaffected by transfection with PMCA4b siRNA and downregulation of PMCA4b expression; representative blot (n = 3). D, Inhibition of PMCA4b expression abrogates protective effect of renalase peptides for HK-2 cells exposed to cisplatin: HK-2 cells exposed to 20μM cisplatin for 24 hrs; cell survival is depicted as % change in survival compared to that in cisplatin-treated HK-2 cells without renalase peptides; cell survival measured by the WST-1 method, peptide concentration 15 μg/ml, indicated in top line; n = 4, * = p<0.05. E, Endogenous expression of PMCA4b in WT and renalase KO mice, western immunoblot using an anti-renalase monoclonal antibody; 10 μg protein loaded in each lane.

We next sought to determine if inhibition of PMCA4b activity modulated renalase mediated MAPK signaling. Caloxin1b, a peptide inhibitor of PMCA4b [15], abrogated renalase-mediated ERK and p38 MAPKs phosphorylation in HK-2 cells (Fig 5A). Since Caloxin1b is reported to also inhibit PMCA1, additional evidence regarding PMCA4b’s role in renalase mediated signaling was obtained by specifically down-regulating PMCA4b expression using siRNA. In control studies, non-targeting siRNAs affected neither PMCA4b expression nor RP-220 mediated p38 (Fig 5B, left panel). In contrast, PMCA4b-targeting siRNAs decreased protein expression by more than 90% and prevented RP-220 mediated p38 phosphorylation (Fig 5B, middle and right panels). The specificity of the interaction between RP-220 and PMCA4b was examined by testing if PMCA4b downregulation also affected epidermal growth factor (EGF) mediated MAPK activation. As shown in Fig 5C, inhibition of PMCA4b expression had no effect on EGF dependent ERK, p38 and JNK phosphorylation.

Down-regulation of PMCA4b expression abrogated the protective effect of RP-220 and RP-H220 against cisplatin cytotoxicity (Fig 5D). We previously showed that in marked contrast to what’s observed in WT mice, neither renalase nor RP-220 is effective at activating MAPK signaling and at protecting renalase KO mice against mild or severe ischemic AKI [13]. Based on these results, we hypothesized that the interaction of RP-220 with its cognate receptor was disrupted in the renalase KO, perhaps due to a decrease in receptor gene or protein expression brought about by deletion of the renalase gene. PMCA4b gene expression in the renalase KO mouse was measured by quantitative PCR, and found to be 11.4 fold lower than in WT control (n = 6, p<0.03). Expression of PMCA4b protein in WT and renalase KO kidneys was evaluated by western immunobloting. As shown in Fig 5E (representative blot), PMCA4b expression in the renalase KO was 63.5±7.5% lower than in WT (n = 6, p<0.03). These data provide strong support for the hypothesis that PMCA4b functions as a renalase receptor, and is critical in mediating the signaling and cytoprotective effects of renalase.

## Discussion

We have identified the plasma membrane calcium ATPase isoform, PMCA4b as a receptor for extracellular renalase. Endogenously expressed PMCA4b and renalase are colocalized at the plasma membrane of HK-2 cells, and the renalase peptide RP-220 can be cross-linked to PMCA4b. Inhibition of PMCA4b enzymatic activity by caloxin1b and down-regulation of PMCA4b expression with siRNA completely abrogated renalase and RP-220-mediated MAPK signaling and cytoprotection.

**Fig 6:**
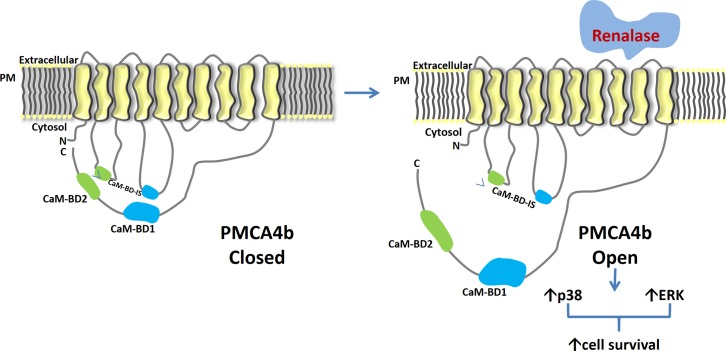
Molecular basis of renalase’s action: working model. PMCA4b: plasma membrane calcium ATPase isoform 4b; PM: plasma membrane; N: amino terminus; C: carboxy terminus; CaM-BD1: calmodulin binding domain 1; CaM-BD2: calmodulin binding domain 2; CaM-BD-IS: calmodulin binding domain interaction sites.

The molecular events that are set in motion by the binding of extracellular renalase to PMCA4b ultimately lead to MAPK activation, and since the data indicate that renalase activates PMCA4b, we propose the working model shown in Fig 6. PMCA4b has previously been characterized as a plasma membrane ATPase involved in cell signaling and cardiac hypertrophy [16]. It ejects Ca2+ from the cytosol of a eukaryotic cell to the external environment using one molecule of ATP in transporting one molecule of Ca2+. It is a P-type ATPase encoded by four genes (ATP2B1–4), the transcripts of which undergo different types of alternative splicing. It appears to regulate local rather than bulk calcium concentration in both excitable and non-excitable cells and to participate in a macromolecular complex that signals through Ras and the MAPKs (24–26). For instance, it modulates bone mass, cardiac contractility and hypertrophy by regulation of NF-kB, neuronal nitric oxide synthase (nNOS), and calcineurin, respectively [17–20]. That these processes are calcium-dependent provides support for the hypothesis that PMCA4b regulates local calcium concentration, thereby altering the activity of its many interacting partners. For example, PMCA4b modulates Ras signaling and ERK activation through its interaction with the tumor suppressor RASSF1, a Ras effector protein involved in H-Ras-mediated signaling [21]. Extracellular renalase and renalase peptides (RP-220) activate the Ras-Raf-MEK-ERK (MAPK) pathway, and perhaps RASSF1 may play a role in RP-220 dependent MAPK signaling.

Our data indicate that the early activation of the stress kinase p38 pathway, and not ERK pathway, is crucial in mediating the protection of renalase against cisplatin cytotoxicity. Stress signals activate MAPKs, which in turn coordinate the cellular responses leading to cell death or survival [22]. While ERK activation by extracellular growth factors tends to promote cell survival, this effect has been shown to be cell and stimuli dependent. The p38 and JNK pathways, which are generally linked to cell death, are activated by a variety of stresses, including oxidants, UV irradiation, and inflammatory cytokines [22]. Cisplatin activates p38, ERK, and JNK in renal epithelial cells.

An unexpected finding in our study is that cells treated with cisplatin and renalase peptide, chemical inhibition of p38 completely eliminated the peptide’s protective effect. Since renalase peptide is associated with early activation of p38, the most likely explanation relates to the biphasic nature of p38 activation by stressful stimuli. For instance, in the presence of tumor necrosis factor (TNF), p38 and JNK are activated twice, first before apoptosis and later at the time of apoptosis [23]. Inhibition of the early JNK and p38 activation, by chemical means or by expression of dominant negative mutants, increases TNF-induced apoptosis, suggesting that early activation of JNK and p38 is prosurvival.

We have presented evidence that PMCA4b functions as a renalase receptor, and mediates renalase dependent MAPK signaling and cytoprotection. In addition to providing insight into the mechanism of action of extracellular renalase, these findings reveal a previously unrecognized mode of regulation of PMCA4b. They provide additional evidence for the hypothesis that PMCA4b functions as a signaling molecule, and given that PMCA4b modulates the development of cardiac hypertrophy, its interaction with renalase may have particular clinical relevance for patients with hypertension and chronic kidney disease.

## Supporting Information

S1 Methods Protein identification by Liquid chromatography-tandem mass spectrometry. (DOCX)
