# Supplementary material for: Identification of a Receptor for Extracellular Renalase
Source: PLoS One. 2015 Apr 23;10(4):e0122932. doi: 10.1371/journal.pone.0122932 (PMC4407985; doi:10.1371/journal.pone.0122932)
Supplement: S1 Methods — (DOCX) [file pone.0122932.s001.docx]

**Method Supplement**

**Protein identification by Liquid chromatography-tandem mass spectrometry**

Liquid chromatography-tandem mass spectrometry (LC-MS/MS) of tryptic digests of the bands of interest was carried out using a LTQ Orbitrap mass spectrometer (Thermo Scientific) equipped with a Waters nanoACQUITY UPLC system. It uses a Waters Symmetry® C18 180µm x 20mm trap column and a 1.7 µm, 75 µm x 150 mm nanoAcquity™ UPLC™ column (37ºC) for peptide separation. Trapping was done at 15µl/min, with 0.1% formic acid in mater for 1 min. Peptide separation was performed with a linear gradient over 80 minutes at a flow rate of 300 nl/min (0.075% Formic Acid in Acetonitrile)

- Positive mode ionization, Mass range (400-2000), Capillary Temperature of 220 degree C, Spray voltage was set at 2kV, and Tube lens at 105V.

- Top 7 peaks per cycle were targeted for MS/MS fragmentation by CID (Data Dependent mode) at Normalized Collision Energy of 35V for 30ms. Peak isolation window was set at 2Da.

- A dynamic exclusion for the DD mode was set for a duration of 60 sec with repeat duration of 30sec.

Database searching:

- Mascot distiller and the Mascot search algorithm were used for database searching (see http://www.matrixscience.com/ for details)
- The database searched= NCBInr_20130301.fasta
- Taxonomy= Human
- Confidence level set to 95% within the MASCOT search engine for protein hits based on randomness

Criteria for positive identification of proteins:

- 2 or more MS/MS spectra match the same protein entry in the database searched
- matched peptides derive from the type of enzymatic digestion performed on the protein
